# Association of genetic polymorphism of *PC‐1* gene (rs1044498 Lys121Gln) with insulin‐resistant type 2 diabetes mellitus in Punjabi Population of Pakistan

**DOI:** 10.1002/mgg3.775

**Published:** 2019-06-28

**Authors:** Abdullah Abdo Albegali, Muhammad Shahzad, Muhammad Ikram Ullah, Saqib Mahmood, Maryam Rashid

**Affiliations:** ^1^ Department of Pharmacology University of Health Sciences Lahore Pakistan; ^2^ Department of Clinical Laboratory Sciences Jouf University Sakaka Saudi Arabia; ^3^ Department of Human Genetics University of Health Sciences Lahore Pakistan

**Keywords:** genetic association, insulin resistance, Pakistan, *PC‐1* gene, type 2 diabetes mellitus

## Abstract

**Background:**

Insulin resistance (IR), known to reduce the response to insulin action, develops with obesity leading to type 2 diabetes mellitus (T2DM). The *PC‐1* gene has been associated with dyslipidemia, polycystic ovarian disease and T2DM in different regions of the world. The objective of the present study was to investigate the genetic association of *PC‐1* rs1044498 polymorphism with insulin resistance in type 2 diabetes in the Punjabi population of Pakistan.

**Methods:**

This **s**tudy was carried out on 161 healthy controls and 161 patients of T2DM with insulin resistance. Whole blood was collected for DNA extraction and molecular studies. PCR‐RFLP with *AvaII* was performed to determine the genotype in cases and controls. Chi‐square and Hardy Weinberg analyses were carried out. Statistical analysis was performed by SPSS software.

**Results:**

The demographic data of cases and controls showed significant differences for different parameters like glucose, insulin, Homeostatic model assessment‐insulin resistance (HOMA‐IR) and lipid profiles (*p *< 0.000). Different statistical models revealed that all the dominant models were found associated in between alleles for disease risk (*p* < 0.001) while no association of *PC‐1* rs1044498 (K121Q) polymorphism was found with insulin‐resistant parameters in T2DM cases.

**Conclusion:**

Overall, the results indicate that the K121Q polymorphism was not found associated with insulin resistance in type 2 diabetes in a Pakistani Punjabi population. This is the first‐ever report about the genotype of *PC‐1* gene in this population.

## 1 INTRODUCTION

Type 2 diabetes mellitus (T2DM) is part of the metabolic syndrome characterized by the body's inability to produce or respond to insulin. Insulin resistance (IR), strongly related with development of T2DM, along with hypertension and dyslipidemia (impaired lipid levels), is one of the main clustering risk factors of metabolic syndrome (Ansarimoghaddam et al., 2018; Eapen, Kalra, Merchant, Arora, & Khan, 2009; Zafar, Khaliq, Ahmad, & Lone, 2019) and other illnesses of cardiovascular diseases (CVD) (Martins, Majzoub, & Agawal, 2018; Roberts, Hevener, & Barnard, 2013). The prevalence of insulin resistance and the metabolic syndrome is growing, mainly in developing countries and in younger populations with estimate ranging from 10% to 40% (14%–63% in Pakistan) in different developing populations (Ali, Khuwaja, Adnan‐ur‐Rahman, & Nanji, 2012; Ansarimoghaddam et al., 2018; Johnson, 2006).

Unhealthy changes in lifestyle also lead to both insulin resistance and metabolic syndrome. An individual's risk of developing insulin resistance and metabolic syndrome is also determined by genetic factors (Brown & Walker, 2016). The vast majority of loci related to T2DM is primarily associated with insulin secretion and β‐cell function with far fewer variants apparently influencing insulin resistance. There are number of reasons for this, not the least that insulin resistance is strongly linked to obesity, and dissecting out the role of common genetic variation in insulin resistance in the absence of obesity has been problematic (Brown & Walker, 2016).

Plasma membrane glycoprotein 1 (PC‐1) is also known as Ectonucleotide Pyrophosphatase/Phosphodiesterase 1 (ENPP1). It is located on the long arm of chromosome 6 (6q23.2) and encodes for a protein which is one of the factors determining the insulin sensitivity. It is a class‐II transmembrane glycoprotein located both on the plasma membrane and in the endoplasmic reticulum (ER). Although, the role of PC‐1 is not completely understood, it is capable of inhibiting the autophosphorylation of insulin receptor by interacting directly with the insulin receptor α‐subunit, thus preventing subsequent downstream signaling (Johnson, 2006; Pappalardo et al., 2017).

A functional missense nucleotide polymorphism (c.517A>C; chr6: 131851228A>C) in exon 4 of *PC‐1* gene (NM_006208.2) that causes an amino acid change from lysine (K) to glutamine (Q) at codon 121 (K121Q) has been described. In vitro studies have shown that the Q variant of *PC‐1* has a stronger interaction with the insulin receptor than with the K variant and reduces insulin receptor autophosphorylation (Pizzuti et al., 1990). Various genetic polymorphism and risk alleles in this gene have been found associated with coronary heart disease, polycystic ovarian disease, obesity, risk of diabetic kidney disease, coronary artery calcification, ischemic heart disease, and T2DM (Di, Dai, & Zhang, 2018; Li, Pan, Cui, & Li, 2016; Moehlecke et al., 2010; Pappalardo et al., 2017; Rozumenko, Harbuzova, Ataman, & Ataman, 2015; Sortica et al., 2015).

The results of association studies of the K121Q polymorphism in various reports remain conflicting and further investigations are needed to elucidate its exact molecular mechanism. Previous studies documented the association of the Q allele of *PC‐1* (rs1044498) with insulin resistance (Gu et al., 2000; Pizzuti et al., 1990) while other studies demonstrated no association of this allele with the risk of disease (Rasmussen et al., 2000). From the South Asian subcontinent, very few reports have been conducted on the involvement of *PC‐1* risk alleles in disease (Prakash, Mittal, Awasthi, Agarwal, & Srivastava, 2013). Therefore, we investigated the relationship between the K121Q polymorphism and IR in T2DM patients of Punjabi population of Pakistan.

## 2 MATERIAL AND METHODS

### 2.1 Ethical statement

Prior to start of the study, institutional approval was obtained from Research and Ethical Board of University of Health Sciences, Lahore, Pakistan. For the collection of samples, guidelines for human subjects were followed (Puri, Suresh, Gogtay, & Thatte, 2009).

### 2.2 Study population

A total of 161 patients who had been recently diagnosed with T2DM and 161 age and sex‐related healthy controls were recruited during the period of August 2013 to August 2016 from the outdoor clinics of different teaching hospitals of Lahore which is the capital of the Punjab province of Pakistan.

Insulin resistance (IR) was confirmed by applying HOMA‐IR equation according to the American Heart Association guidelines. A total of 5 ml blood sample was obtained from each participant for biochemical and molecular studies.

### 2.3 DNA extraction and genotyping

After blood collection in EDTA tubes, genomic DNA was extracted from the whole blood by using a BioNeer Blood DNA extraction Kit (centrifugal column; Daejeon city, Korea) according to the manufacturer's specifications. Polymerase chain reaction (PCR) was carried out using primers (F‐5'CTGTGTTCACTTTGGACATGTTG3'; R‐5'GACGTTGGAAGATACCAGGTTG3') of *PC‐1* polymorphism (rs1044498), followed by the restriction fragment length polymorphism using *AvaII* (BioLab) restriction enzyme to genotype the alleles. The digested products were resolved on 2.5% agarose gel electrophoresis for band interpretation along with marker DNA (100 bp ladder).

### 2.4 Statistical analysis

SPSS 21.0 was used for data analysis of the quantitative variables by performing Student's *t* test for continuous values and by the chi‐squared test for nominal variables. The genotype distributions, which were expected to be in Hardy–Weinberg equilibrium, were confirmed using a goodness‐of‐fit chi‐square test, and the differences in variables and genotype frequency distributions between the patients and controls were tested using a two‐sided chi‐squared test. The relationship between the *PC‐1* K121Q polymorphism and IR in T2DM was described by calculating odds ratios and 95% CIs through a binary logistic regression analysis. Statistical significance was considered if *p* ≤ 0.05.

## 3 RESULTS

### 3.1 Population characteristics

A total of 161 cases of T2DM and 161 healthy controls comprised the population analyzed in our study. The demographics and clinical parameters of all participants are shown in Tables 1 and 2. The range of age of the participants was 45–65 years. The IR group comprised 82 (50.9%) male patients and 79 (49.1%) female patients. No difference in gender (*p* < 1.00) was observed between cases and controls. An analysis of the body mass index in cases revealed 91 (56.5%) patients were overweight and one (0.3%) patient was obese (Table 1). The clinical parameters including waist circumference, HOMA‐IR, and lipid profile were found to be significantly different in cases when compared to controls (*p* < 0.001) which explain the metabolic abnormalities among IR of T2DM patients (Table 2).

**Table 1:** Demographic data of patients with insulin‐resistant type 2 diabetes and controls

Parameter	Over all	Controls	Cases	χ^2^(*df*)	*p*‐value
	*n* (%)	*n* (%)	*n* (%)		
Age					
≤50 years	181 (56.2)	70 (43.5)	111 (68.9)	21.209 (1)	**<0.001**
>50 years	141 (43.8)	91 (56.5)	50 (31.1)		
Body mass index					
Under weight	06 (1.9)	06 (3.7)	00 (0.0)	123.953 (3)	**<0.001**
Normal weight	222 (68.9)	153 (95.0)	69 (42.9)		
Overweight	93 (28.9)	02 (1.2)	91 (56.5)		
Obese	01 (0.3)	00 (0.0)	01 (0.3)		
Gender					
Males	164 (50.9)	82 (50.9)	82 (50.9)	0.000 (1)	1.00
Females	158 (49.1)	79 (49.1)	79 (49.1)		

Statistically significant values (*p* <0.05).

**Table 2:** Comparison (Mean ± *SD*) of biochemical parameters in patients (T2DM) and controls

Parameter	Controls	Cases	t/U^a^	*p*‐value
Waist Circumference(cm)	77.43 ± 8.7	104.22 ± 8.7	27.479	**<0.001**
FPG (mg/dL)	73.53 ± 9.1	180.13 ± 16.3	72.453	**<0.001**
FPI (uU/mL)	6.29 ± 1.1	37.38 ± 3.8	60.610	**<0.001**
HOMA‐IR	1.16 ± 0.3	32.00 ± 19.9	63.166	**<0.001**
TC (mg/dL)	171.30 ± 8.7	230.84 ± 60.5	4,304.00	**<0.001**
TG (mg/dL)	122.09 ± 12.1	203.12 ± 50.0	825.00	**<0.001**
HDL (mg/dL)	63.32 ± 5.1	38.70 ± 3.3	105.50	**<0.001**
LDL (mg/dL)	210.21 ± 10.9	151.51 ± 61.2	5,017.50	**<0.001**
VLDL (mg/dL)	24.41 ± 2.4	40.63 ± 9.6	820.50	**<0.001**

a The bold values showed the significant *p*‐value between cases and controls. *t* test used for normal distributed data while Mann–Whitney *U* test is used for not‐normally distributed data, Fasting plasma glucose (FPG), Fasting plasma insulin (FPI).

### 3.2 Genetic association studies (*PC‐1* rs1044498, K121Q polymorphism)

**Figure 1:**
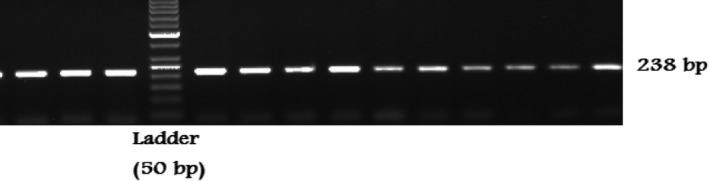
Genotype of *PC‐1* alleles on agarose gel. A band size of 238 bp of PCR products for *PC‐1* K121Q polymorphism after running its PCR product in 2% agarose gel using 50‐bp DNA marker

**Figure 2:**
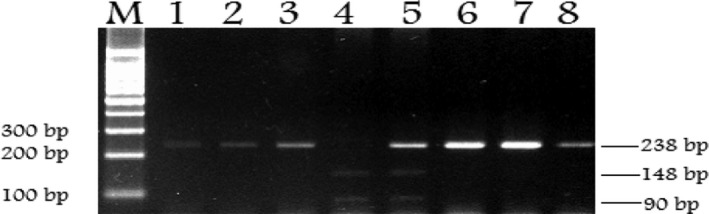
Distribution of genotypes analysis on agarose gel. Restriction fragments of PCR products for *PC‐1* K121Q polymorphism using *AvaII* enzyme in 2.5% agarose gel. Lane M shows 100‐bp DNA marker, lanes 1, 2, 3, 6, 7, and 8 show 238 bp single fragment of KK genotype (wild type homozygous), lane 4 shows 248, 148, and 90 bp fragments of heterozygous KQ genotype, lane 5 shows 148, and 90 bp fragments of rare homozygous QQ genotype

The amplified product of *PC‐1* rs1044498, polymorphism was a band of 238 bps size on a 2.0% gel (Figure 1). Band patterns defined single band of 238 bp for KK genotype (wild type), double bands (148, 90 bp) for QQ genotype (homozygous) and triple bands (238, 148, and 90 bp) defined for KQ (heterozygous) genotype (Figure 2).

To investigate the genetic association between *PC‐1* K121Q polymorphism and IR, the genotype frequency distributions of IR cases and controls were analyzed. The genotype frequencies of the *PC‐1* K121Q polymorphism were 95.0% (KK), 4.3% (KQ), and 0.6% (QQ) in the cases and 80.8% (KK), 18.0% (KQ), and 1.2% (QQ) in the controls (Table 3).

**Table 3:** Genotype models for disease association of *PC‐1* rs1044498 SNP genotype

Genotypes/models	Controls	Cases	*p‐*Value	OR crude [95% CI]	*p‐*Value adjusted^a^	OR Adjusted^a^ [95% CI]
	*n* (%)	*n* (%)				
Genotypes						
Codominant model						
KK (Lys/Lys) WT homozygous	130 (80.8)	153 (95.0)	**<0.001**	1.00	0.16	1.00
KQ (Lys/Gln) heterozygous	29 (18.0)	07 (4.3)		**0.21 (0.09–0.48)**		0.07 (0.00–2.38)
QQ (Gln/Gln) homozygous	02 (1.2)	01 (0.6)		0.42 (0.04–4.74)		0.00 (0.00‐NA)
Dominant model						
KK	130 (80.8)	153 (95.0)	**<0.001**	1.00	0.055	1.00
KQ and QQ	31 (19.2)	08 (5.0)		**0.22 (0.10–0.49)**		0.07 (0.00–2.36)
Recessive model						
KK and KQ	159 (98.8)	160 (99–4)	0.56	1.00	0.89	1.00
QQ	02 (1.2)	01 (0.6)		0.50 (0.04–5.53)		0.00 (0.00‐NA)
Over dominant						
KK and QQ	132 (82.0)	154 (95.7)	**<0.001**	1.00	0.056	1.00
KQ	29 (18.0)	07 (4.3)		**0.21 (0.03–0.49)**		0.07 (0.00–2.38)
Log‐additive						
‐—	—	—	**<0.001**	**0.27 (0.13–0.57)**	0.055	0.07 (0.00–2.37)

a Bold values showed the significant difference of *p*‐value (0.05) and odd ratio between patients and controls. Adjusted for: Age, Gender, BMI, HOMA, TC, TG, HDL, LDL, and VLDL.

After adjustment for age, gender, BMI, HOMA, TC, TG, HDL, LDL, and VLDL, the relationship between the *PC‐1* K121Q polymorphism and IR was explored. In dominant model, it was found that KQ/QQ genotypes of *PC‐1* K121Q polymorphism contributed to only 19.2% occurrence of insulin resistance (OR = 0.22, 95% CI = 0.10–0.49, *p* < 0.001 and after adjustment OR = 0.07 95% CI = 0.00–2.36, *p *= 0.055).

## 4 DISCUSSION

The association between insulin resistance and *PC‐1* rs1044498 (K121Q) polymorphism was analyzed in our case–control investigation. This study is the first to examine *PC‐1* K121Q polymorphism in insulin‐resistant T2DM patients from Pakistan. The functions of PC‐1 protein remain to be fully elucidated, however, it has been demonstrated that overexpression of PC‐1 suppresses activity of insulin receptor of tyrosine kinase and subsequently cellular signaling in various cells (Maddux & Goldfine, 2000).

In this study, the demographic data showed that there was a correlation of insulin resistance with other biochemical parameters like glucose, insulin, and serum lipid profiles which were significantly different in the cases and controls. A genetic association between the *PC‐1* K121Q polymorphism and insulin resistance in T2DM was not established in our study. Consequently, it may be proposed that *PC‐1* K121Q combined KK/QQ alleles are not risk alleles to arbitrate the disease risk in T2DM patients.

The *PC‐1* K121Q polymorphism is a functional missense alteration (Sortica et al., 2015) in which the 121Q variant binds insulin receptors more firmly than the 121K variant (Costanzo et al., 2001). Previous studies have documented an association of this polymorphism with insulin resistance, T2DM, and metabolic syndrome (Abate et al., 2003; Bhatti et al., 2010; Daoming, Chaoren, Xijun, & Junjun, 2006; Grarup et al., 2006; Marchenko et al., 2018; Vasudevan, Ismail, Ali, & Mansor, 2009). On the other hand, some other studies did not document the genetic association of *PC‐1* K121Q polymorphism with insulin resistance (Rasmussen et al., 2000). A study conducted in the China also showed that the genotype distribution of *PC‐1* K121Q polymorphism has no association with T2DM or any of metabolic syndrome characters (Daoming et al., 2006). These results are similar to the present study which have not established any association of *PC‐1* with risk of type 2 diabetes in the Pakistani Punjabi population.

A meta‐analysis on the correlation between *PC‐1* K121Q polymorphism and T2DM risks showed positive findings and established the genetic associations (Abate et al., 2005; Hamaguchi et al., 2004), that the exact molecular mechanism of risk factor for this gene is still unclear.

In conclusion, our study did not find any association between insulin resistance type 2 diabetes mellitus and *PC‐1* K121Q polymorphism. It is suggested that further studies on larger scale are to be carried out to explain the precise molecular mechanism of association between studied SNP and development of insulin resistance in the pathogenesis type 2 diabetes mellitus.

## CONFLICT OF INTEREST

The authors declare that they have no competing interests.

## AUTHOR CONTRIBUTIONS

MS and SM: conceived and design the study. AAA: collected the samples from subjects and performed laboratory experiments. AAA, MIU, and MR: data integration, statistical analyses, and its interpretation. AAA and MIU: drafted the manuscript. MS, SM, and MR: edited and approved the manuscript.
